# Mixed Layer Depth Trends in the Bay of Biscay over the Period 1975–2010

**DOI:** 10.1371/journal.pone.0099321

**Published:** 2014-06-12

**Authors:** Xurxo Costoya, Maite deCastro, Moncho Gómez-Gesteira, Fran Santos

**Affiliations:** EPHYSLAB, Environmental PHYsics LABoratory, Facultad de Ciencias, Universidad de Vigo, Ourense, Spain; University of Aveiro, Portugal

## Abstract

Wintertime trends in mixed layer depth (MLD) were calculated in the Bay of Biscay over the period 1975–2010 using the Simple Ocean Data Assimilation (SODA) package. The reliability of the SODA database was confirmed correlating its results with those obtained from the experimental Argo database over the period 2003–2010. An iso-thermal layer depth (TLD) and an iso-pycnal layer depth (PLD) were defined using the threshold difference method with ΔT = 0.5°C and Δσ_θ_ = 0.125 kg/m^3^. Wintertime trends of the MLD were calculated using winter extended (December-March) anomalies and annual maxima. Trends calculated for the whole Bay of Biscay using both parameters (TLD and PLD) showed to be dependent on the area. Thus, MLD became deeper in the southeastern corner and shallower in the rest of the area. Air temperature was shown to play a key role in regulating the different spatial behavior of the MLD. Negative air temperature trends localized in the southeastern corner coincide with MLD deepening in this area, while, positive air temperature trends are associated to MLD shoaling in the rest of the bay. Additionally, the temperature trend calculated along the first 700 m of the water column is in good agreement with the different spatial behavior revealed for the MLD trend.

## Introduction

Upper Ocean is characterized for a quasi-homogeneous layer where temperature, salinity and density scarcely vary with increasing depth [24]. This homogeneity layer is caused by turbulent vertical mixing that is driven by heat loss from the ocean to the atmosphere, as well as by wind stress. The deepest layer affected by this turbulent mixing is called mixed layer depth (MLD), which marks the width of the upper ocean that interacts with the atmosphere. MLD presents a high spatial and temporal variability. Thus, it varies at different temporal scales, from diurnal [3] to decadal scales [7] and from mesoscale to large spatial scale [8], [36]. The deepest MLDs in the North Atlantic occur in winter and early spring. During spring the onset of surface heating produces a restratification of the upper ocean, giving as result shallower MLDs, especially during summer. These variations of the MLD, that reach hundreds of meters over the year, have important biological implications [13], [47].

MLD plays an important role in influencing the ocean's role in air-sea interaction. Thus, it is fundamental in the exchange of heat and freshwater between the ocean and the atmosphere and influences other processes such as the formation of water masses or the activity of phytoplankton. [11] analyzed the influence of the winter MLD in the concentrations of nutrients and dissolved inorganic carbon in the Bay of Biscay. They confirmed that a deeper MLD as a consequence of a warmer winter produced higher surface winter concentrations of nutrients and dissolved inorganic carbon. Moreover, they related this fact with an enhancement of the spring bloom in the Bay of Biscay. Similar results have been reported in the North Atlantic Ocean [2], [20].

Long-term MLD variability has been analyzed at some fixed moorings in the North Atlantic [34], [37]. Several studies on MLD variability have been carried out using global databases. Thus, for example, [25] used the World Ocean Atlas and [7] used the World Ocean Database. In fact, the main problem to develop a MLD long-term variability study is the lack and scarcity of temperature and salinity profiles to analyze the subsurface ocean variables, especially for salinity profiles [8]. After the development of the International Argo Project [1] the coverage of temperature and salinity profiles has been increased strikingly all over the world. However, its recent deployment prevents the realization of long term studies.

Taking in account the limitations commented above, it is necessary to find alternative databases to study subsurface ocean variables such as MLD at a regional scale. Some authors [4], [5], [6] have developed a project called Simple Ocean Data Assimilation (SODA). SODA is based on a method that uses an ocean model in conjunction with data assimilation providing a useful background of information beneath the sea surface. This fact allows to obtain a complete view of the different hydrographic processes that take place in the area, both at global [6], [51] and at basin scale [14], [18], [39], [52] which is the aim of the present study focused in the Bay of Biscay.

The Bay of Biscay is a semi-enclosed area located in the northeastern Atlantic Ocean between 0–10°W and 43–48°N. The oceanic part of the Bay of Biscay is influenced by subpolar and subtropical Atlantic gyres and it is characterized by following an anti-cyclonic, weak (1–2 cm/s) and variable circulation [26], [44]. A distinctive feature is the wide of the continental shelf along the French coast, especially the northern area, ranging from 60 to over 200 km and with a very gentle slope of 0.12% [38]. In contrast, the southern coast is characterized by a narrower continental shelf (7–20 km wide). Various studies have analyzed sea surface temperature trends for different periods and locations in the Bay of Biscay [10], [15], [16], [27], [40]. Furthermore, salinity and temperature variability beneath the sea surface were studied, focusing over the Eastern North Atlantic Central Water that is the main water observed at the upper layers in the Bay of Biscay [18], [19], [32]. In addition, [46] analyzed MLD variability using a one-dimensional water column model (GOTM) with data from NCEP/NCAR reanalysis over the period 1948–2008. They found that during 1970s and 1980s, MLDs were strikingly shallower than from 1995 onwards.

The aim of the present study is to analyze the trend of the MLD during wintertime for the whole Bay of Biscay over the period 1975–2010. The relevance of this study is the possibility to know whether MLD trends are similar at any location within the Bay of Biscay or there exist important spatial differences. This analysis provides complementary information respect to previous studies, which have analyzed the long-term MLD variability taking data from a particular area of the Bay of Biscay [46]. For this purpose two complementary datasets have been used. Namely, Argo is based on in situ measurements and SODA combines simulation and assimilated data. A comparison between them was carried out in order to confirm the reliability of SODA dataset. The used datasets and methods are described in section 2. Two different approaches were considered to calculate the wintertime MLD: the temperature criterion to define the isothermal layer depth (TLD) and the density criterion to define the iso-pycnal layer depth (PLD). This fact, together with the calculation of two different wintertime parameters (winter anomalies and annual maxima) gives as result a complete analysis of the wintertime MLD trends. Section 3 shows the results and the discussion. This includes an analysis of the atmospheric forcing carried out correlating air temperature, wind stress and precipitation minus evaporation trends with MLD results. Moreover, temperature trends along the water column (0–700 m) were also considered to be linked with MLD. Finally, section 4 compiles the main conclusions.

## Datasets and Methods

Two databases were used to obtain temperature and salinity data of the upper ocean in the Bay of Biscay. On the one hand, the “Simple Ocean Data Assimilation” (SODA) package (http://dsrs.atmos.umd.edu/), which was built following an ocean model based on Geophysical Fluid Dynamics Laboratory MOM2 physics was used [4], [5]. Assimilated data includes temperature and salinity profiles from the World Ocean Atlas-94 [28], as well as additional hydrography, sea surface temperature [42], and altimeter sea level from the ERS-1, Geosat, and TOPEX/Poseidon satellites. Reanalysis of world ocean climate variability are available from 1958 to 2010 at monthly scale, with a horizontal spatial resolution 0.5° x 0.5° and a vertical resolution of 40 levels, ranging from 5 m to more than 5000 m decreasing the resolution with depth. The long coverage period of this dataset allows focusing the study on the period 1975–2010, when the higher temperature increasing was detected in the North Atlantic region [10], [12], [16], [17], [23], [27], [35].

On the other hand, the database derived from the International Argo Project was used [1]. Argo data were extracted from the US Global Ocean Data Assimilation Experiment (USGODAE) server (http://www.usgodae.org/argo/argo.html), which is one of the two Argo Global Data Assembly Center (GDAC) that stored Argo float data. Under this project, floats started to be deployed in 2000, increasing deployments year on year, until reaching more than 3500 active floats all over the world in 2013. This number of floats is enough to attain part of the goals that the Argo Project had when it was designed, such as a global resolution of 3°x3°. This amount of floats produces more than 100.000 profiles each year because they are designed to generate a profile each 10 days. Overall, profiles reach a maximum depth of 1500–2000 meters with higher resolution at the uppermost layers. The accuracy of pressure, temperature and salinity measurements is ±2.4dbar, ±0.005°C and ±0.01psu, respectively [1]. The recent onset of the Argo Program and the progressive deployment of Argo floats since 2002 involve that the number of profiles for the first years of the Argo Program in the Bay of Biscay were limited. In fact, there were not valid profiles for some months in 2003 and 2004.

Apart from these two datasets, meteorological large-scale reanalysis data of temperature, precipitation, latent heat and wind speed above sea surface were extracted from the National Center for Environmental Prediction/National Center for Atmospheric Research (NCEP/NCAR) website (www.cdc.noaa.gov). Data are supplied on a T62 Gaussian grid corresponding to a 1.9° both in latitude and longitude and monthly time resolution from 1948 on. Air temperature was considered at 2 m from surface, while wind was regarded at 10 m. Evaporation was calculated dividing the heat flux loss by the latent heat of water (2.5x10^6^ J kg^−1^) following [19]. Moreover, wind speed was used to calculate wind stress according to the formula: τ_wind_  =  ρ_air_C_D_ U_h_^2^, where C_D_ is the wind-drag coefficient (0.0011), ρ_air_ is the air density (1.2250 kg m^−3^ at 15°C) and U_h_ is the wind speed above sea surface (10 m).

The Spearman rank correlation coefficient was used to analyze the significance of trends and correlations due to its robustness to deviations from linearity and its resistance to the influence of outliers [49].

The method used to determine the MLD was the threshold difference method that is based in the choice of a temperature or density threshold value. MLD is determined as the depth where the temperature or density exceeds the threshold value with respect to a reference depth located near the sea surface. The threshold difference method was shown to be more stable than the gradient method based on temperature or density gradients [5]. Hence, an important point is the choice of the threshold value. There is not a consensus about what are the best threshold values, which is related to the fact that MLD features change both in space and in time due to the different physical processes and hydrographic features that characterize the ocean all over the world (see [8], [21], [24], [33] for recent discussions). Taking in account these studies, the most commonly used threshold values (ΔT = 0.5°C and Δσ_θ_ = 0.125 kg/m^3^) were selected for potential temperature and density (e.g. [36]).

Argo and SODA datasets allow taking advantage of their salinity and temperature vertical resolution. A potential temperature threshold to define the iso-thermal layer depth (TLD) and a potential density threshold to define the iso-pycnal layer depth (PLD) were considered following the terminology applied by [22] who analyzed different procedures to calculate MLDs using Argo floats. In this way we can detect possible biases derived of the salinity effect on the MLD that can produce important differences between TLD and PLD (e.g., [8], [22]). Potential values were calculated to avoid pressure problems with increasing depth following the method describe in [48]. Besides, a reference depth of 15 m was chosen to prevent the diurnal oscillations of temperature and the effect of precipitation and evaporation that takes place in the first meters beneath sea surface [41]. This choice was adopted because the first two levels in the SODA database are 5 m and 15 m.

As it was previously mentioned, the deepest MLDs occur during winter and early spring in the North Atlantic region (e.g. [25], [36]). For this reason, the present study is focused on the analysis of winter MLD. Thus, annual maxima MLD and winter extended (December-March) anomalies were calculated.

**Figure 1:**
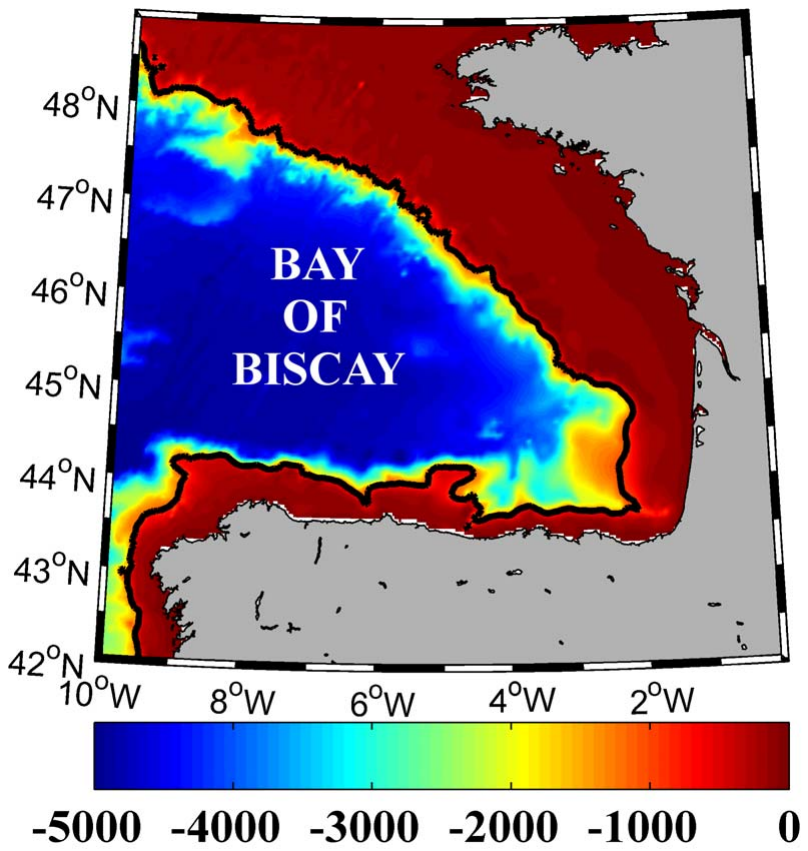
Bay of Biscay bathymetry (m). The isobath corresponds to -1000 m.

First of all, both databases were compared for the period 2003–2010 in order to know the reliability of SODA database. To carry out this comparison, all available data among 2003–2010 in the Bay of Biscay (43.25–48.25°N//0.25–9.75°W) were extracted. In this case, the domain was slightly enlarged, with respect to the rest of the study, in order to include a larger number of Argo profiles, especially during the years 2003 and 2004 when the number of profiles was limited. Thus, a better sampling was obtained ensuring better results in the comparison. As we mentioned above, this area is characterized by its wide continental shelf, especially along the French coast (Figure 1). For this reason, to ensure that depth does not influence TLD/PLD calculations only profiles deeper than 1000 m were selected. A total of 101 points carried out the requirements in the SODA database, while a total of 3362 profiles, which corresponds to 58 floats, were extracted from the ARGO database. These 3362 profiles were submitted to a quality control selecting only profiles with a control flag of 1, following the Argo quality control manual v2.8 [50] (http://www.argodatamgt.org/Documentation) what is enough to guarantee the quality of data. Argo and SODA profiles were interpolated for each meter following a linear interpolation in order to get the most accurate TLD/PLD value.

Data from the 101 points of SODA database and good quality profiles from ARGO database were horizontally averaged for the whole region and for each month over the period 2003–2010. Thus, an average profile was obtained for each month and for each database. Then, TLDs and PLDs were calculated based on each monthly averaged profile. Finally, annual maxima and winter anomalies were determined and correlated for both datasets. An annual maximum corresponds to the winter month when TLD/PLD attains the highest depth. TLP and PLD winter anomalies were calculated considering the available profiles for the December-March months and subtracting to each month, the mean value of that month for the whole period of time.

The area between 43.75–47.25°N and 2.25–8.25°W was selected to analyze trends in wintertime MLD over the Bay of Biscay for the period 1975–2010. Only grid points deeper than 1000 m were considered, which includes 70 points from SODA database. The methodology was described above but TLDs/PLDs were calculated in this case for each of the 70 points in order to know if trends are similar in the whole bay. Trends were assumed to be linear and obtained by fitting time series (TLDs and PLDs) to a straight line in a least-squares sense. Finally, annual maxima and winter anomalies trends of TLD/PLD were correlated with atmospheric variables (air temperature, P-E balance and wind stress).

## Results and Discussion

**Figure 2:**
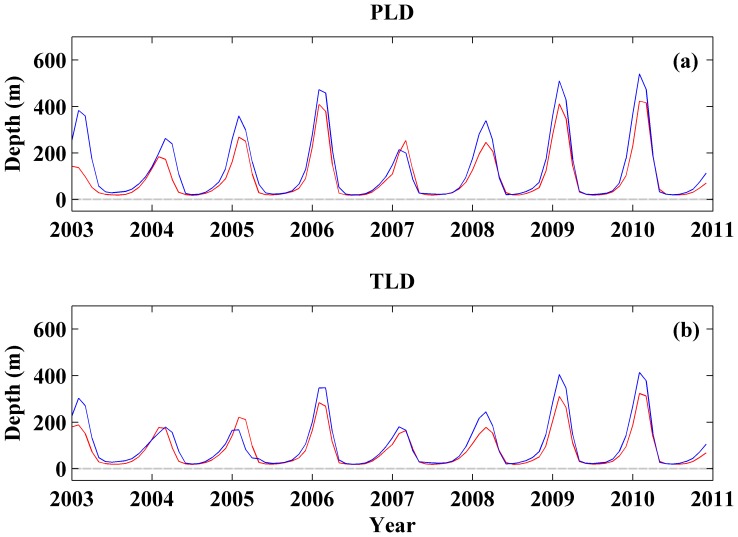
Comparison between Argo and SODA databases. (a) Isopycnal layer depth and (b) isothermal layer depth variability using Argo database (blue line) and SODA database (red line). A 1-2-1 filter was used to smooth signals only for visualization.

The correlation between Argo and SODA using annual maxima and winter anomalies is shown in Table 1. Correlation coefficients are slightly higher for winter anomalies (0.74 for PLD and 0.71 for TLD) than for annual maxima (0.66 for PLD and 0.64 for TLD). All these correlation coefficients are significant at a level higher than 90%. In addition, monthly averaged PLDs and TLDs for both databases are shown in Figure 2 over the period 2003–2010. Overall, a good agreement was observed between both databases for the whole period. Nevertheless, SODA (red line) underestimates the MLD depth both for TLD (2003, 2006, 2008, 2009 and 2010) and PLD (all years except 2007). We should note that PLD depends both on temperature and salinity and so the sources on uncertainty are higher compared to TLD. The largest differences were observed in winter 2003. This fact seems to be related to the few Argo profiles available during that year. The Argo program was launched in 2002, which caused a less uniform sampling in the bay over the first years.

**Table 1:** Correlations between annual maxima and winter anomalies of PLD/TLD calculated from ARGO and SODA databases for the period 2003–2010.

	Annual Maxima	Winter anomaly
PLD	0.66 (0.074)	0.74 (0.037)
TLD	0.64 (0.089)	0.71 (0.046)

Threshold values of ΔT = 0.5°C and Δσ_θ_ = 0.125 kg/m^3^ were used for potential temperature and for potential density, respectively. Statistical significance (p value) is showed in brackets.

**Figure 3:**
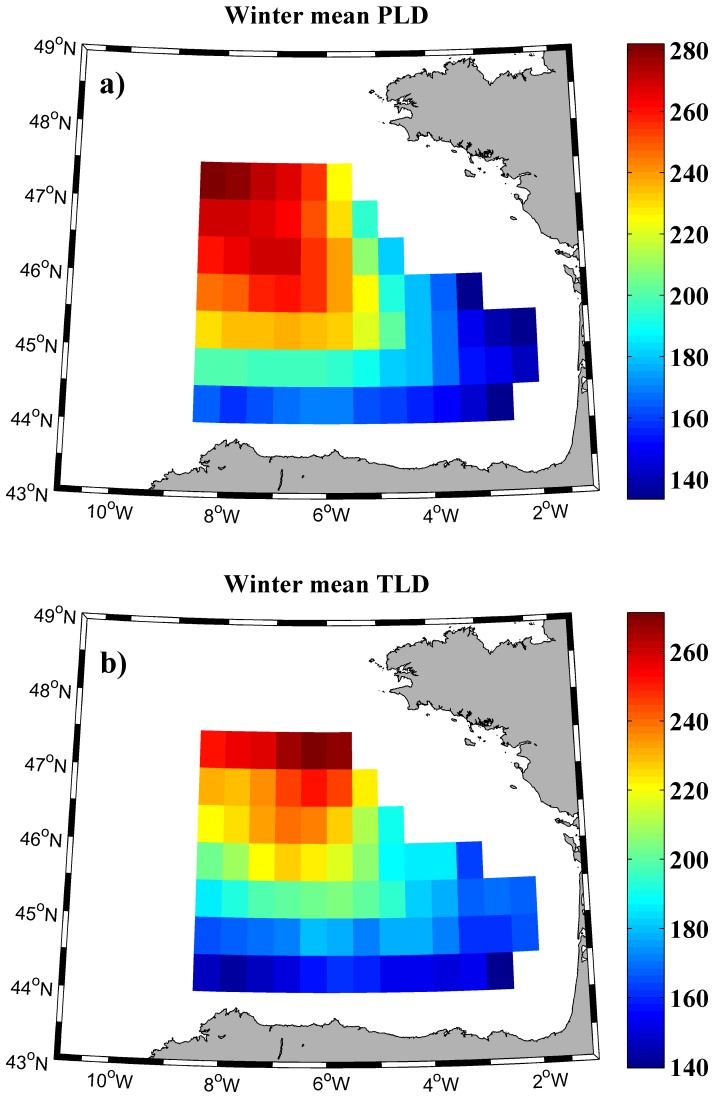
Mean values of winter mixed layer depth. (a) Winter mean isopycnal layer depth (m) and (b) winter mean isothermal layer depth (m) over the period 1975–2010.

Once the reliability of SODA database was confirmed using in-situ measurements, it was used to characterize the MLD in the bay over the period 1975–2010. Winter PLD/TLD means were calculated for each of the 70 grid points that constitute the bay (Figure 3). A remarkable southeast-northwest MLD gradient can be observed in both frames. In this way, the deepest mean MLDs, around 270 m, were found near the northern boundary while the shallowest mean MLDs, around 140 m, were noticed at the southeastern corner. Overall, not significant differences were found between winter mean PLD and winter mean TLD. The observed differences are related to haline forcing, which is a phenomenon produced by different oceanic processes such as surface freshwater fluxes or freshwater advection. These changes can produce that the halocline is located above the thermocline or vice versa. This effect on MLD has been previously described all over the world by different authors [9], [31].

**Figure 4:**
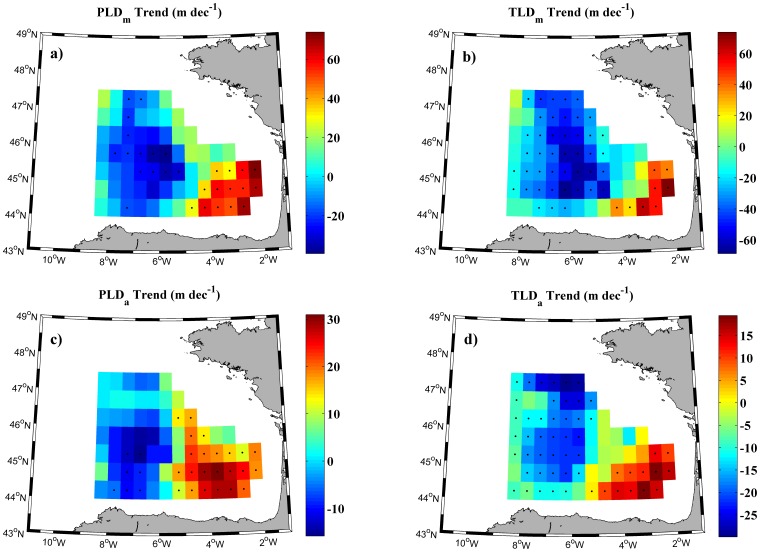
Trend values of mixed layer depth. (a) (b) Annual maxima trends and (c) (d) winter anomalies trends (m dec^−1^) of PLD (left) and TLD (right) in the Bay of Biscay for the period 1975–2010. Black dots represent points with a significance level higher than 90%.

Long-term trends of annual maxima and winter anomalies of TLD and PLD for each grid point in the Bay of Biscay for the period 1975–2010 are shown in Figure 4. Black dots represent points with statistical significance higher than 90%. Negative (positive) trends mean that MLD tends to be shallower (deeper). In all cases, the most important feature is that points located at the southeastern corner of the Bay of Biscay show a significant positive trend. MLD deepens in this zone and shallows in the rest of the area, especially at the central part.

As it was previously mentioned, annual maxima correspond to the month when TLD/PLD attain their maximum, which corresponds to winter or early spring (commonly March) in the Bay of Biscay. First, trends in annual maxima of MLD (Figure 4a,b) will be analyzed. Trends at grid points with significant positive values are observed to range from 20 to 60 m per decade, being higher for PLD (Figure 4a) than for TLD (Figure 4b). In a similar way, trends at grid points with significant negative values range from -10 m to -60 m per decade for TLD and are around -25 m per decade for PLD. The spatial distribution of significant positive and negative trends is similar for winter anomalies (Figure 4c,d). In this case, positive MLD trends range from 5 to 25 m per decade and negative trends from -5 to -25 m. Differences between TLD and PLD can also be observed for winter anomalies as previously noticed for annual maxima. The positive MLD trend described above for the southeasthern of the bay is in good agreement with results obtained by [46]. They found that MLD was shallower during the 1970s and 1980s, becoming deeper from 1995 onwards.

**Figure 5:**
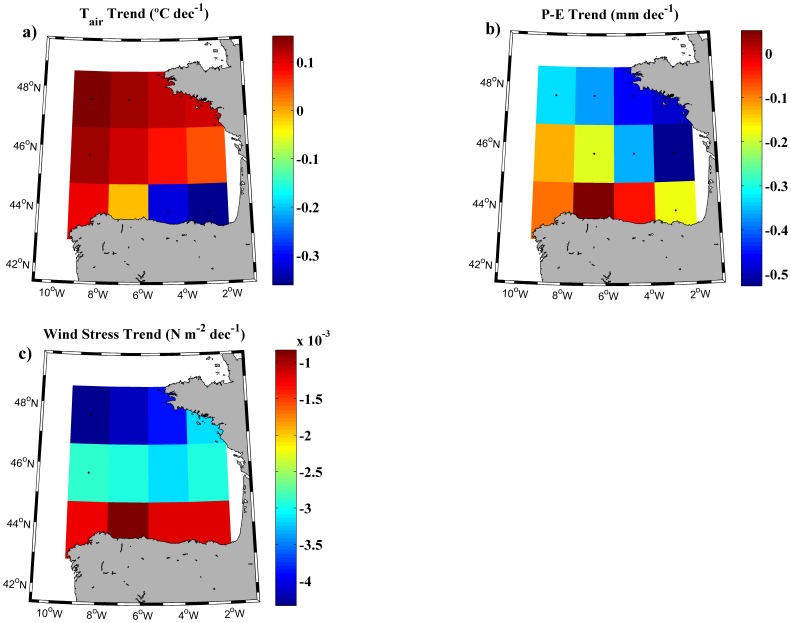
Trends of atmospheric variables. (a) Air temperature trend (°C dec^−1^), (b) P-E (precipitation minus evaporation) balance trend (mm dec^−1^) and (c) wind stress trend (N m^−2^dec^−1^) calculated for the whole Bay of Biscay over the period 1975–2010. Black dots represent points with a significance level higher than 90%.

In order to know the influence of the atmospheric forcing over the MLD trend, long-term trends calculated using the temperature and density criterion were correlated with different atmospheric variables (air temperature, P-E balance and wind stress) for the period 1975–2010. Trends, which were assumed to be linear, were calculated for these atmospheric variables following the same procedure used for PLDs and TLDs (Figure 5).

Only the air temperature trend shows a different sign depending on the area of the Bay of Biscay (Figure 5a), being positive over most of the bay, with values around 0.1°C per decade. However, negative trends, with maximum values of −0.3°C per decade, were detected at the southeastern corner. Dots represent trends with significance higher than 90%. Hence, the air temperature trend seems to be spatially consistent with annual maxima and winter anomalies TLD/PLD trends shown in Figure 2. P-E balance (Figure 5b) shows a significant negative trend, with differences between -0.5 mm per decade in the northeast boundary and around -0.15 mm per decade in central and southeastern boundary. Regarding wind stress (Figure 5c), grid points show negative trends for the whole area, being higher in the north (-0.004 N m^−2^ per decade) than in the south (-0.001 N m^-2^ per decade) of the bay.

As we mentioned above, trends in air temperature seem to be correlated with trends in MLD (TLD and PLD). To analyze the significance of this resemblance between patterns we have proceeded as follows. Considering, for example, TLD as an ocean variable, it was spatially averaged for all grid points where the variable was observed to have significant positive trends (>90%). This resulted in a one-dimensional signal (TLD^+^), where only a value is stored per month. The same protocol was followed for the grid points with significant decreasing trends (TLD^−^ signal). On the other hand, a similar signal was created for air temperature, in such a way that Tair^+^ (Tair^−^) was obtained by averaging the grid points with significant positive (negative) trends. The protocol previously described for TLD was also followed for PLD.

Table 2 shows the correlation between ocean variables (TLD and PLD) and Tair. As it previously mentioned the areas where air temperature decreases (increases) coincide macroscopically with the areas where MLD increases (decreases). Thus, only the comparisons among ocean and atmospheric variables with different sign were considered. MLD^+^ signals were only compared with Tair^−^ signals and vice-versa. Significant negative correlations were obtained between air temperature and TLD/PLD. This means that increasing air temperature results in the decrease of TLDs/PLDs depths. All correlations show a significance level higher than 95%. In particular, correlations between the Tair^−^ and PLD^+^/TLD^+^ (both correspond to the southeast corner of the Bay of Biscay) range from −0.35 for winter anomalies of TLD to −0.48 for annual maxima. Higher correlation coefficients were found between Tair^+^ and PLD^−^/TLD^−^, with values of −0.69 for winter anomalies of PLD and −0.83 for annual maxima. The significance of correlations proves the fact that could be foreseen by visual inspection, namely, decreasing MLD trends are related to increasing in air temperature and vice-versa. On the other hand, correlations with P-E and wind stress did not show significant results for any of the possible combinations.

**Table 2:** Left, correlations between points of negative air temperature trends (T_air_^−^) and points of positive annual maxima (PLD_m_/TLD_m_) and winter anomalies (PLD_a_/TLD_a_) trends.

	T^−^_air_		T^+^_air_
PLD^+^_m_	−0.45 (0.007)	PLD^−^_m_	−0.7 (0.001)
TLD^+^_m_	−0.48 (0.004)	TLD^−^_m_	−0.83 (0)
PLD^+^_a_	−0.39 (0.019)	PLD^−^_a_	−0.69 (0.001)
TLD^+^_a_	−0.35 (0.035)	TLD^−^_a_	−0.8 (0)

Right, correlations between points of positive air temperature trends (T_air_^+^) and points of negative annual maxima (PLD_m_/TLD_m_) and winter anomalies (PLD_a_/TLD_a_) trends. Statistical significance (p value) is showed in brackets.

The influence of air temperature on MLD was inferred previously at the southeastern Bay of Biscay by [45]. They analyzed an extreme mixing event, characterized for a deeper than normal MLD in 2005. They found that winter 2005 was characterized by a relatively high sensible heat flux (the highest since 1965) which is a variable where air temperature plays a crucial role. Air temperature decrease causes an increase in net heat loss and this is directly associated with MLD sinking. Moreover, [46] indicated that winter net heat losses are also related to storminess activity. This fact agrees with results presented in Figures 5b since the balance between precipitation and evaporation (P-E) has a negative trend in most of the bay. On the other hand, wind stress does not play an important role in winter mixed layer formation in the Bay of Biscay according to these authors. Therefore, mixed layer development in the bay shows to be mostly conducted by convection processes than by wind stress.

Considering a global scale, this study shows that a relative small and semi-enclosed sea such as the Bay of Biscay may show opposite trends related to MLD variability. This fact is due to the high dependence of MLD on local atmospheric forcing and hydrographic conditions. For this reason, results shown in this study cannot be extrapolated to other regions. However, it seems that atmospheric forcing can play a similar role to that described for the Bay of Biscay in neighboring areas. Thus, [7] found that MLD variability is not closely related to variation in local wind speed in the North Atlantic (60W–30W, 35N–45N) for the period 1960–2004. However, these authors found that wind speed plays an important role to explain MLD variability in the North Pacific (180W–150W, 35N–45N) for the same period.

**Figure 6:**
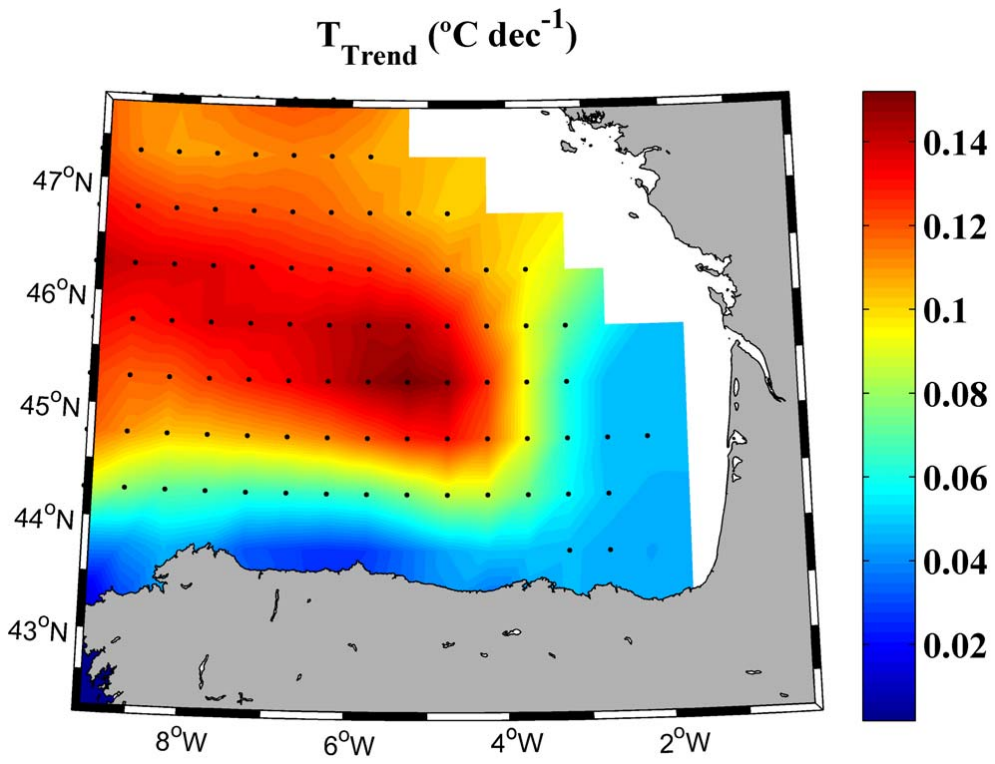
Winter ocean temperature trend for to the upper 700–2010. Black dots represent grid points with a significance level higher than 95%.

Winter temperature trend for the upper 700 m of the water column was also analyzed over the period 1975–2010 using SODA database (Figure 6). According to [29], [30] the most important variations in the heat content during the last decades occurred in the upper 700 m. This is also shown for the Iberian Peninsula [43] and the Bay of Biscay [18]. The whole area presents a positive trend but with different intensity. Warming is higher at the northwest corner and in the central part of the bay with values ranging from 0.1–0.15°C per decade. However trends are almost negligible at the south and at the southeastern corner with values around 0.04°C per decade. Macroscopically, the area of higher (negligible) warming coincides with the area where MLD has become shallower (deeper).

## Conclusions

The present study analyzes wintertime MLD trends in the Bay of Biscay over the period 1975–2010, which coincides with the highest warming period ever detected in the North Atlantic. MLD was calculated in two different ways, namely, by using a potential temperature criterion to define the TLD and a potential density criterion to define the PLD. Time evolution of MLD was analyzed in terms of trends in winter anomalies and in annual maxima.

MLD tended to deepen at the southeastern corner and shallow at the rest of the bay. Correlations carried out between atmospheric variables and MLD trends evidence that air temperature plays a key role in regulating the MLD variability. In this way, negative air temperature trends, localized in the southeastern part of the bay, are related with MLD deepening at this area and positive air temperature trends observed for the rest of the bay are related to MLD shoaling. MLD evolution did not show significant correlations with wind stress and precipitation minus evaporation. Finally, warming calculated for the upper 700 m is observed to be more intense at the area where MLD became shallower and negligible at the area where MLD deepened.

The MLD analysis carried out in this study highlights that mesoscale features can play an important role even in small semi-enclosed areas like the Bay of Biscay, where the observed behavior is far from being spatially homogeneous. Most of the research previously conducted in the area was focused on local events which do not necessarily represent the overall behavior of the Bay.

The complex bathymetry of the area and the fact that the bay is surrounded by land in more than 50% of its perimeter give rise to important local variations in the oceanic and atmospheric variables. Thus, the inner part of the Bay, which is much shallower than the central one, is highly dependent on continental inputs. This is especially important for temperature, which is influenced by land masses in winter and summer and by the presence of mountain ranges like *Picos de Europa* that can modify the passage of fronts in winter.
